# Auxin-induced AsARF16 complex orchestrates lncRNA125175-mediated ceRNA networks to regulate garlic somatic embryogenesis

**DOI:** 10.1093/hr/uhag016

**Published:** 2026-01-20

**Authors:** Yunhe Bai, Jiaojiao Ruan, Fangling Jiang, Fei Ding, Yuqing Cui, Min Liu, Ping Li, Meng Zhang, Mengqian Li, Hanyu Wei, Rong Zhou, Zhen Wu

**Affiliations:** Sanya Research Institute of Nanjing Agricultural University, College of Horticulture, Nanjing Agricultural University, Nanjing 210095, China; Sanya Research Institute of Nanjing Agricultural University, College of Horticulture, Nanjing Agricultural University, Nanjing 210095, China; Sanya Research Institute of Nanjing Agricultural University, College of Horticulture, Nanjing Agricultural University, Nanjing 210095, China; Sanya Research Institute of Nanjing Agricultural University, College of Horticulture, Nanjing Agricultural University, Nanjing 210095, China; Sanya Research Institute of Nanjing Agricultural University, College of Horticulture, Nanjing Agricultural University, Nanjing 210095, China; Sanya Research Institute of Nanjing Agricultural University, College of Horticulture, Nanjing Agricultural University, Nanjing 210095, China; College of Agricultural and Environmental Sciences, University of Georgia, Athens, GA 30602, USA; Sanya Research Institute of Nanjing Agricultural University, College of Horticulture, Nanjing Agricultural University, Nanjing 210095, China; Sanya Research Institute of Nanjing Agricultural University, College of Horticulture, Nanjing Agricultural University, Nanjing 210095, China; Sanya Research Institute of Nanjing Agricultural University, College of Horticulture, Nanjing Agricultural University, Nanjing 210095, China; Sanya Research Institute of Nanjing Agricultural University, College of Horticulture, Nanjing Agricultural University, Nanjing 210095, China; Sanya Research Institute of Nanjing Agricultural University, College of Horticulture, Nanjing Agricultural University, Nanjing 210095, China; Department of Food Science, Aarhus University, Agro Food Park 48, 8200 Aarhus, Denmark; Sanya Research Institute of Nanjing Agricultural University, College of Horticulture, Nanjing Agricultural University, Nanjing 210095, China

## Abstract

Somatic embryogenesis is a crucial biotechnological approach for effectively addressing garlic variety degeneration and improving yield and quality. Previous studies have demonstrated that the long noncoding RNA 125175 (lncRNA125175) is specifically induced and expressed during somatic embryogenesis, and may act as an endogenous target mimic of AsmiR393h to participate in the regulation of somatic embryogenesis. On this basis, the present study systematically elucidated the functions of the lncRNA125175/AsmiR393h/*AsTIR1* regulatory module and its upstream transcriptional mechanism. First, transient expression assays in tobacco leaves and protoplast experiments in garlic suggested that lncRNA125175 served as a competing endogenous RNA (ceRNA) to sequester AsmiR393h, thereby attenuating its post-transcriptional cleavage of the target gene *AsTIR1*. Promoter analysis revealed that all core components of this module contain auxin *cis*-acting elements, and the promoter activities of lncRNA125175 and *AsTIR1* are significantly induced by exogenous auxin, suggesting that this ceRNA network is precisely regulated by auxin signaling. Further weighted gene co-expression network analysis identified the auxin response factor AsARF16 as a key upstream regulator. Yeast one-hybrid and two-hybrid assays indicated that AsARF16 can directly bind to the promoter of lncRNA125175, and interact with the transcription factor AsWRKY31 and the auxin signaling factor AsIAA33 to form a transcriptional activation complex. In conclusion, this study uncovers a cascade pathway from auxin signal perception (the AsARF16 complex) to transcriptional activation (lncRNA125175), followed by post-transcriptional ceRNA regulation. It systematically clarifies the molecular mechanism underlying its precise regulation of garlic somatic embryogenesis, providing a critical theoretical basis for the targeted improvement in garlic regeneration efficiency and genetic transformation systems.

## Introduction

Garlic (*Allium sativum* L.), belonging to the genus *Allium* in the Liliaceae family, is a globally important herbaceous plant with remarkable agricultural, medicinal, economic, and industrial value, as its unique flavor compounds and health-care properties have laid a solid foundation for its widespread application [1, 2]. However, its sterility and long-term asexual propagation via bulbs have led to a series of severe challenges such as variety degradation, low propagation coefficient, and accumulation and spread of diseases, especially viral diseases, which seriously restrict the improvement in yield and quality as well as the breeding of new varieties [3, 4]. Somatic embryogenesis, featuring genetic stability, high propagation efficiency, and experimental reproducibility, is regarded as an indispensable tool in modern plant biotechnology and a key approach to fundamentally break through the breeding bottlenecks of such asexually propagated crops [5]. This technique enables the efficient regeneration of complete plants from a single somatic cell, and has been widely applied in plant germplasm conservation, high-quality seedling production, artificial seed development, and molecular breeding, serving not only as the technical core for virus elimination and rejuvenation, cryopreservation of germplasm resources, and efficient micropropagation, but also as an indispensable recipient system for modern molecular breeding operations such as genetic transformation and gene editing [6]. Therefore, deciphering and optimizing the regulatory mechanism of garlic somatic embryogenesis are of great theoretical significance and urgent practical demand.

Among the numerous regulators of somatic embryogenesis, auxin plays a crucial role in promoting the dedifferentiation of somatic cells into embryonic callus and inhibiting the germination of somatic embryos [7]. 2,4-Dichlorophenoxyacetic acid (2,4-D) is a commonly used auxin analog that exerts its function via its receptor TRANSPORT INHIBITOR RESPONSE1/AUXIN SIGNALING F-box PROTEIN (TIR1/AFB), an F-box protein that triggers the ubiquitin-dependent degradation of auxin/indole-3-acetic acid (Aux/IAA) transcriptional repressors [8–10]. Consequently, auxin response factors (ARFs) are released to transcriptionally regulate the expression of downstream auxin-responsive genes. However, the efflux of 2,4-D is transiently inhibited by the 1-*N*-naphthylphthalamic acid (NPA). NPA disrupts somatic embryogenesis by interfering with PIN-FORMED (PIN) protein-mediated auxin transport [11, 12]. Collectively, the coordinated actions of 2,4-D, TIR1, and NPA orchestrate a precise regulatory framework essential for plant regeneration.

miRNAs, which are 20–24 nucleotides (nt) in length, regulate gene expression by complementary binding to mRNAs at the post-transcriptional level [13]. A growing body of research demonstrates that miRNAs play important roles in somatic embryogenesis [14–16]. In *Dimocarpus longan* Lour., dlo-miR408-3p promotes cell division and differentiation during early somatic embryogenesis by targeting *DlNUDT23* to regulate riboflavin biosynthesis and m^6^A modification [17]. Similarly, the role of miR827 in phosphorus homeostasis and sucrose accumulation was uncovered during somatic embryogenesis in *Solanum betaceum* Cav. [18]. More recently, the highly conserved miR393 family has been reported to function in plant somatic embryogenesis. miR393 plays a key role in auxin signaling through its targeting of *TIR1/AFB* genes, which are critical for auxin perception and the activation of downstream responses [19]. The expression of miR393h was accumulated, resulting in low expression of *TIR1* and *AUXIN F-BOX PROTEIN2* (*AFB2*) at the early stage of *Arabidopsis* somatic embryogenesis [20]. In addition, miR393h modulates somatic embryogenesis by downregulating *TIR1* and *AFB2* [21].

Long noncoding RNAs (lncRNAs) have recently emerged as key regulators of gene expression [22]. LncRNA contains microRNA (miRNA) recognition elements, which can weaken the inhibitory effect of miRNA on their target genes [23]. This mechanism is called competing endogenous RNAs (ceRNA), which are implicated in diverse biological processes [24–26]. Plant endogenous target mimics (eTM) were first discovered in *Arabidopsis thaliana*: the lncRNA *IPS1* harbors a three-nucleotide bulge between the 9th and 10th bases at the 5′ end of its miR399-binding region, which inhibits the cleavage of target mRNAs by miR399 and thereby mediates the response to low-phosphorus stress [27]. In tomato, lncRNA39896 and lncRNA40787 act as an eTM of miR166b and miR394, respectively, and regulate resistance to pathogenic bacteria [28, 29]. In cotton, lncRNA354 functions as an eTM of miR160b, indirectly regulating ARFs induced under salt stress [30]. In terms of somatic embryogenesis, the miR390–tasiRNAs–ARF network has been shown to regulate somatic embryogenesis in longan [31]. In total, 5110 lncRNAs are identified in coconut embryogenic callus, and the relationship between lncRNA, miRNA, and messenger RNA (mRNA) are predicted [32]. Despite these advances, the regulatory mechanisms of lncRNA-mediated networks in garlic somatic embryogenesis remain largely unexplored.

During plant somatic embryogenesis, transcription factors from multiple families such as WRKYGQK (WRKY), Homeodomain–Leucine Zipper (HB), and basic Helix–Loop–Helix (bHLH) are specifically induced [33]. Their functions are primarily involved in core processes, including hormone signaling, stress response regulation, and embryonic morphogenesis. In *Arabidopsis*, phosphorylated AtWRKY2 protein activates the expression of the *AtWOX8*, thereby regulating embryonic development [34]. Meanwhile, WRKY23 indirectly influences *Arabidopsis* embryo development by mediating auxin signal transduction [34]. In *Larix principis-rupprechtii*, LpWRKY65 binds to the W-box element in the promoter of the high-mobility group box (*LpHmgB10*), significantly enhancing its transcriptional activity [35]. The high expression of *LpHmgB10* improves intracellular reactive oxygen species scavenging capacity, ultimately promoting somatic embryogenesis and development in *L. principis-rupprechtii* [35]. Furthermore, in various species such as hybrid sweetgum, longan, and Panax ginseng, members of the WRKY transcription factor family have been identified to participate in the regulation of somatic embryogenesis [36–38].

In our previous study, strand-specific RNA sequencing was performed on key stages of garlic somatic embryogenesis, leading to the systematic identification of differentially expressed long noncoding RNAs (lncRNAs) and microRNAs (miRNAs) [39]. Through bioinformatics analysis, we constructed a candidate ceRNA regulatory network, wherein a highly induced lncRNA (lncRNA125175) was predicted to act as an eTM for AsmiR393h. It may regulate the expression of its target gene *TIR1* by competitively sequestering AsmiR393h. However, the regulatory relationships, potential functions, and upstream regulatory mechanisms among the components of this network remain unclear. Here, we aim to address this gap and investigate whether lncRNA125175 modulates *AsTIR1* expression through competitive bonding to AsmiR393h and its potential molecular regulatory mechanism influencing the process of garlic somatic embryogenesis. To achieve the goal, this study constructed the lncRNA125175–AsmiR393h–*AsTIR1* ceRNA regulatory module via comprehensively employed multiple molecular techniques. We further explored the responsive characteristics of this ceRNA regulatory module to auxin signaling, deciphered the interaction mechanism between AsIAA33 and *AsTIR1*, and clarified the regulatory effect of the AsARF16-AsWRKY31 transcriptional regulatory complex on the transcriptional expression of lncRNA125175. Through these investigations, this study systematically revealed the multi-level post-transcriptional regulatory mechanism of ‘auxin signaling-transcription factor complex-lncRNA-miRNA-target gene’ during garlic somatic embryogenesis. This work will not only enrich the theoretical knowledge of the molecular regulatory network underlying plant somatic embryogenesis, but also provide an important theoretical basis and potential molecular regulatory targets for optimizing garlic tissue culture systems, improving somatic embryo induction efficiency, and promoting garlic variety improvement.

## Results

### lncRNA125175 was noncoding RNA

**Figure 1:**
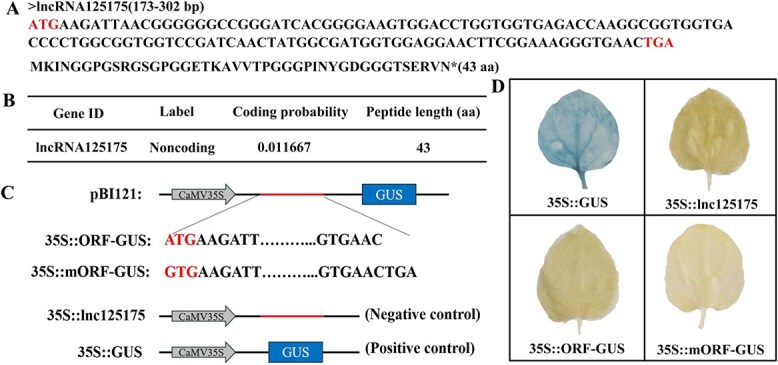
Analysis of potential coding for lncRNA125175. (A) lncRNA125175 polypeptide sequence. (B) Coding potential analysis of lncRNA125175 using CPC2 program. (C) Schematic structure of recombinant vector: *35S::ORF-GUS*, deletion of termination codon (TGA); *35S::mORF-GUS*, mutation of initiation codon ATG to GTG; *35S::lncRNA125175* was used as negative control; *35S::GUS* was used as positive control. (D) GUS staining results after transient injection of tobacco leaves.

The full-length sequence of lncRNA125175 was obtained by 5′/3′ RACE (rapid amplification of cDNA ends) (Fig. S1). To determine whether lncRNA125175 was a noncoding RNA, the CPC2 online tool (https://cpc2.gaolab.org/) was applied to verify its coding ability. LncRNA125175 was predicted to have no protein-coding potential but contained an open reading frame (ORF) sequence that encodes 43 amino acids (Fig. 1A and B). We further identified the coding ability of lncRNA125175 and found that *35S::ORF-GUS* and *35S::mORF-GUS* showed no activity, which proved that lncRNA125175 was an ncRNA (Fig. 1C and D). The GUS gene was not expressed after the transient transformation of the *35S::ORF-GUS* fusion vector in tobacco, indicating that the ORF of lncRNA125175 has no coding ability (Fig. 1C and D).

### A ceRNA regulatory mechanism among lncRNA125175, AsmiR393h, and *AsTIR1*

**Figure 2:**
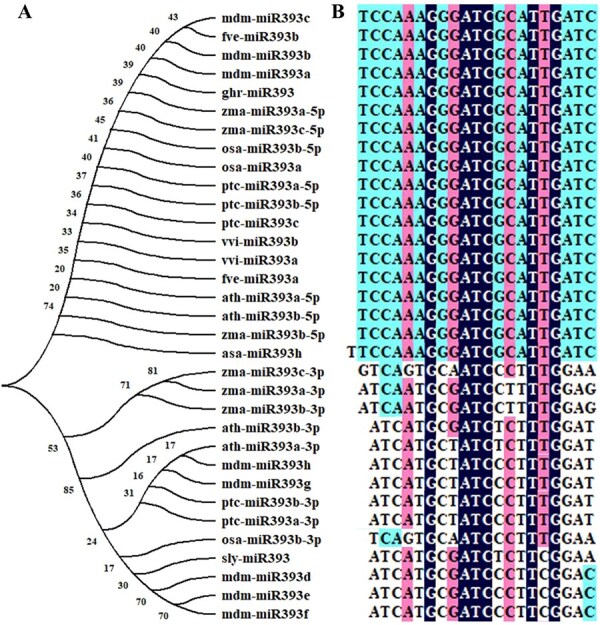
(A) Evolutionary analysis and (B) sequence alignment of miR393h. Ath, *Arabidopsis thaliana*; osa, *O. sativa*; mdm, *M. domestica*; sly, *S. lycopersicum*; fve, *Fragaria vesca*; ghr, *G. hirsutum*; ptc, *P. trichocarpa*; vvi, *Vitis vinifera*; zma, *Z. mays*.

Our previous study identified lncRNA125175, AsmiR393h, and AsTIR1 as key regulators of garlic somatic embryogenesis [39]. Here, the secondary structure of the *AsMIR393h* precursor was predicted using RNAfold (http://rna.tbi.univie.ac.at//cgi-bin/RNAWebSuite/RNAfold.cgi) (Fig. S2). The MEGA-X software (https://www.megasoftware.net/) was applied to analyze the evolution of the miR393 family precursor sequences and the mature sequence alignment, involving 10 plant species such as *A. thaliana*, *Solanum lycopersicum*, *Oryza sativa*, and *Gossypium hirsutum*. The number of miR393 family members differed among species: only one member in tomato, cotton, and garlic, whereas four members in *A. thaliana*, five members in *Populus trichocarpa*, six members in *Zea mays*, eight members in *Malus domestica*, etc. The mature sequence of the miR393 family was highly consistent in the 5′ arm branch based on the alignment of the evolutionary map and the mature sequence (Fig. 2A and B).

**Figure 3:**
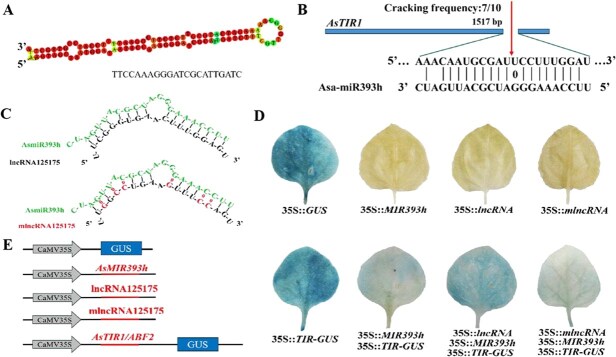
(A) Secondary structure prediction of AsmiR393h precursor gene, (B) AsmiR393h cleavage site for *AsTIR1*, (C) schematic diagram of lncRNA point mutation site, (D) tobacco transient expression validating that lncRNA can act as an eTM for AsmiR393h, and (E) regulatory relationship verification vector construction diagram.

Further characterization revealed that the mature AsmiR393h was embedded within a complete stem-loop structure, predominantly being localized at the 5′ end of the stem arm (Fig. 3A). Using 5′ RLM-RACE, we tested that AsmiR393h directed the cleavage of AsTIR1 transcripts between the 10th and 11th nucleotides at the 5′ end of AsmiR393h, unequivocally identifying *AsTIR1* as a direct target of AsmiR393h (Fig. 3B). To validate the regulatory relationships among lncRNA125175, AsmiR393h, and *AsTIR1*, we constructed four GUS reporter vectors (Fig. 3C and E). The *35S::lncRNA125175*, *35S*::*mlncRNA125175*, *35S*::*MIR393h*, and *35S*::*AsTIR1-*GUS were individually or co-transformed into *Nicotiana benthamiana*. The co-expression of *35S::MIR393h* with *35S::AsTIR1-*GUS led to a significant reduction in GUS activity compared to the expression of *35S::AsTIR1*-GUS alone, demonstrating that AsmiR393h negatively regulated *AsTIR1* expression through transcript cleavage (Fig. 3D). When the three GUS vectors *35S::lncRNA125175*, *35S::MIR393h*, and *35S*::*AsTIR1*-GUS were co-transformed into tobacco, the staining area of leaves was greater than the transformation of *35S*::*MIR393h* and *35S*::*AsTIR1*-GUS (Fig. 3D). When we transferred vector *35S*::*mlncRNA125175*, *35S*::*MIR393h*, and *35S*::*AsTIR1*-GUS, the staining area of tobacco was consistent with transforming *35S*::*MIR393h* and *35S::AsTIR1-*GUS (Fig. 3D). Taking all together, lncRNA125175 functioned as an eTM of AsmiR393h, thereby attenuating its cleavage activity on *AsTIR1* and contributing to the ceRNA regulatory network during somatic embryogenesis in garlic.

### Protoplast analysis of lncRNA125175/AsmiR393h/*AsTIR1* transient expression

**Figure 4:**
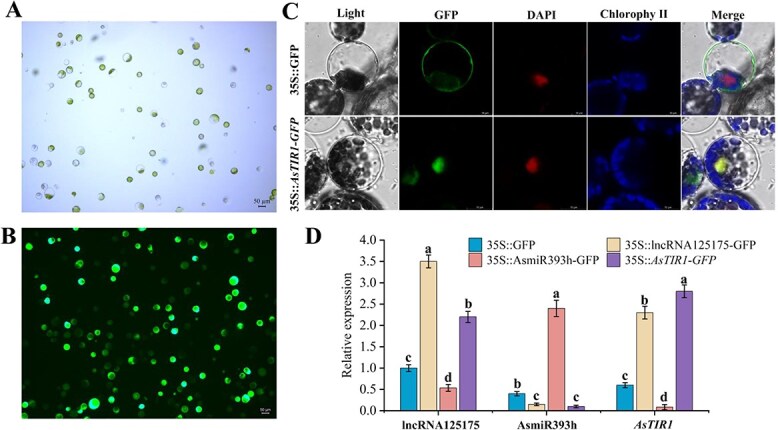
Transient transformation protoplast analysis. (A) Protoplast extraction. (B) Protoplast FDA staining. (C) *35S::lncRNA125175-GFP*, *35S::AsmiR393h-GFP*, *35S::AsTIR1-GFP*, *35S::GFP* transiently translocated protoplasts and subcellular localization. (D) RT-qPCR analysis of lncRNA125175, AsmiR393h, and *AsTIR1*.

Protoplasts were isolated from the leaves of ‘*Ershuizao*’ garlic, the viability of which was tested using fluorescein diacetate (FDA) staining (Fig. 4A and B). To investigate the subcellular localization and regulatory relationships among lncRNA125175, AsmiR393h, and *AsTIR1*, we performed transient overexpression in these protoplasts and observed fluorescence after overnight culture (Fig. 4C). While GFP fluorescence from the *35S::*GFP control was detected in both cell membrane and nucleus, the *35S::AsTIR1-*GFP signal was specifically localized to the nucleus. By contrast, no fluorescence was observed for either *35S::lncRNA125175-*GFP or *35S::AsMIR393h-*GFP (Fig. 4C). Subsequent reverse transcription-quantitative polymerase chain reaction (RT-qPCR) analysis revealed that overexpression of lncRNA125175/*AsTIR1* reduced AsmiR393h expression, whereas overexpression of AsmiR393h downregulated both lncRNA125175/*AsTIR1* transcript levels (Fig. 4D).

### LncRNA125175–AsmiR393h–*AsTIR1* regulated callus formation by responding to 2,4-D

**Figure 5:**
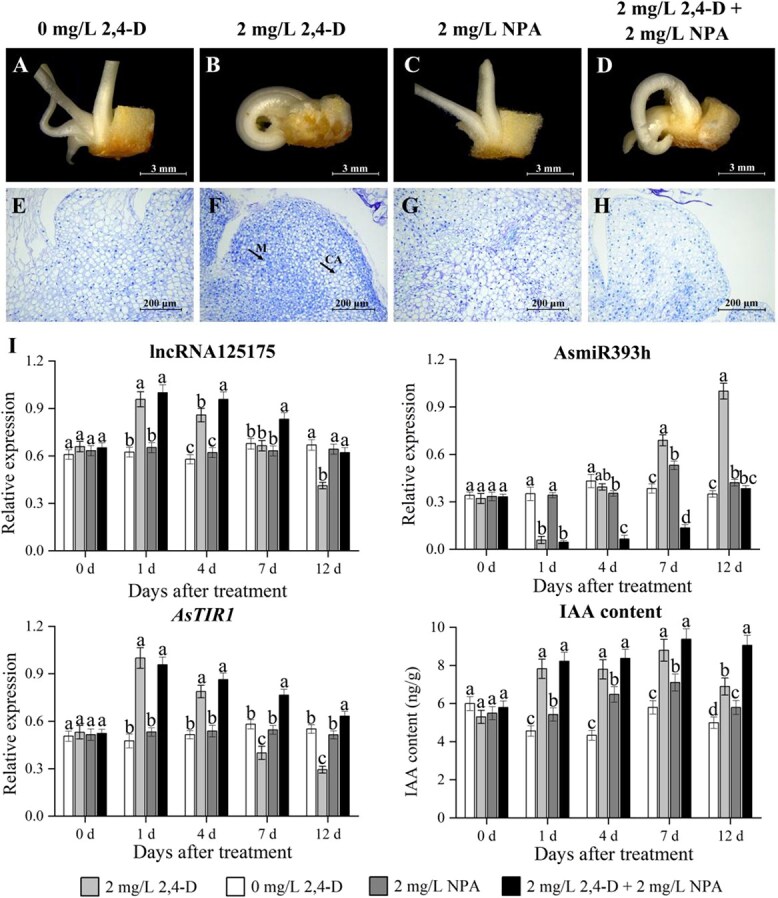
Effects of 2,4-D and NPA on callus formation. (A, B, C, D) The appearance of 0 mg/l 2,4-D, 2 mg/l 2,4-D, 2 mg/l NPA, and 2 mg/l 2,4-D + 2 mg/l NPA after 12 days, respectively. (E, F, G, H) The tissue structures induced by 0 mg/l 2,4-D, 2 mg/l 2,4-D, 2 mg/l NPA, and 2 mg/l 2,4-D + 2 mg/l NPA for 12 days, respectively. M, meristem; CA, callus cells. (I) Changes in lncRNA-AsmiR393h-*TIR1* gene expression under 2,4-D and NPA treatment.

Given the established regulatory roles of auxin and the *TIR1/AFB* pathway in plant somatic embryogenesis, we treated garlic explants with 2,4-D (an auxin analog) and NPA (an auxin transport inhibitor) to investigate the function of the lncRNA125175–AsmiR393h–*AsTIR1* module. On 12 days of treatment, bulb discs in 0 mg/l 2,4-D and 2 mg/l NPA treatment group only produced buds, with no callus formation observed in their external morphology (Fig. 5A and C). Although cells in the 2 mg/l NPA treatment group varied in size and exhibited irregular arrangement, they did not form the loose cell clusters characteristic of callus tissue based on paraffin section analysis (Fig. 5G). After 12 days of treatment with 2 mg/l 2,4-D, the bulb discs showed significant enlargement and developed yellow callus on the surface (Fig. 5B). Paraffin sections indicated the presence of densely arranged meristematic cell clusters (M) in this group, accompanied by callus cells (CA) formed through cellular expansion, which exhibited smaller or less distinct nuclei (Fig. 5F). In the 2 mg/l 2,4-D + 2 mg/l NPA treatment group, granular protrusions appeared at the base of the bulb discs. Paraffin sections revealed loosely structured, lightly stained, and transparent cells, displaying characteristics of early-stage cell division during callus formation (Fig. 5H).

Gene expression analysis of the lncRNA125175–AsmiR393h–*AsTIR1* module revealed dynamic responses to auxin (Fig. 5I). The lncRNA125175 and *AsTIR1* expression displayed a declining trend, while AsmiR393h levels increased under 2 mg/l 2,4-D treatment, which was consistent with their competitive binding relationship. Notably, the expression of lncRNA125175 and *AsTIR1* was significantly lower in the group being co-treated with 2 mg/l 2,4-D and 2 mg/l NPA than that treated with 2 mg/l 2,4-D alone after 4 days of treatment. This suggested that elevated auxin levels led to a more pronounced suppression of the lncRNA125175–AsmiR393h–*AsTIR1* pathway when being combined with the disruption of polar auxin transport. These findings highlighted the auxin-responsive nature of the lncRNA125175–AsmiR393h–*AsTIR1* regulatory network during callus formation and somatic embryogenesis in garlic.

### *AsARF16* regulated callus formation by binding to lncRNA125175 promoter

**Figure 6:**
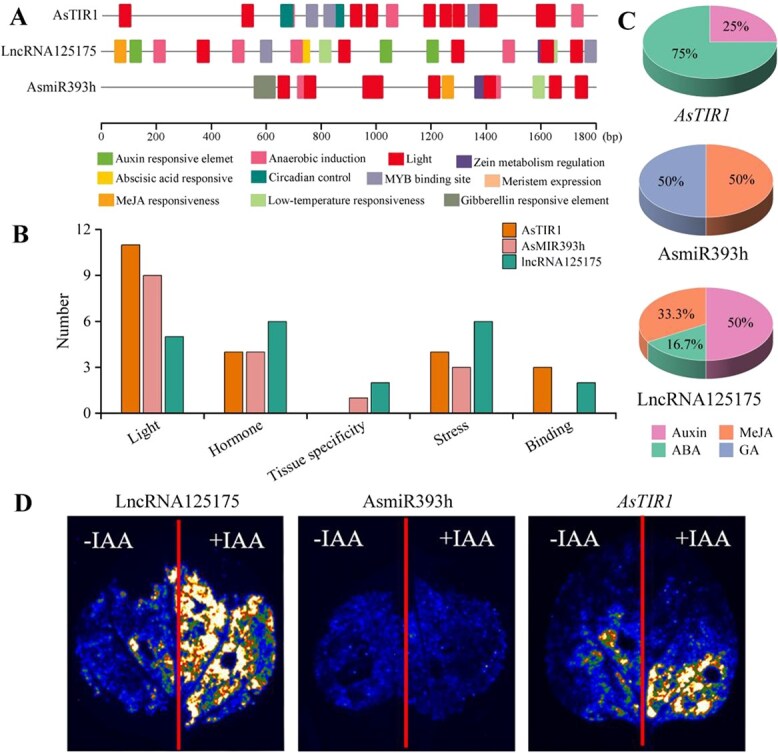
Analysis of lncRNA125175/AsmiR393h/*AsTIR1* gene promoter. (A) The distribution of *cis*-acting elements in 1800-bp promoter region upstream of lncRNA125175, AsmiR393h, and *AsTIR1*. (B) Classification and quantity of *cis*-acting components. (C) Statistics of hormone-related elements of action. (D) lncRNA125175/AsmiR393h/*AsTIR1* gene promoter activity and response to exogenous auxin result.

To elucidate the auxin-responsive regulatory mechanism of the lncRNA125175–AsmiR393h–*AsTIR1* module, we performed comprehensive *cis*-acting element analysis of the 1800-bp promoter regions upstream of lncRNA125175, AsmiR393h, and *AsTIR1*. Bioinformatic analysis identified 11 functionally significant *cis*-regulatory elements, such as light-responsive, hormone-responsive, tissue-specific, stress-related, and transcription factor binding site elements (Fig. 6A and B). lncRNA125175, AsmiR393h, and *AsTIR1* contained a large number of hormone-related elements, including auxin, GA_3_, ABA, and MeJA (Fig. 6C). Additionally, transient expression of the LUC reporter genes in tobacco leaves was performed to further detect the promoter activity of lncRNA125175, AsmiR393h, and *AsTIR1*. LUC verification system was further applied to transiently express pro^lncRNA125175^::pGreenII0800-LUC, pro^AsmiR393h^::pGreenII0800-LUC, and pro*^AsTIR1^*::pGreenII0800-LUC in tobacco leaves. The promoter activities of lncRNA125175 and *AsTIR1* were enhanced after 2,4-D was applied (Fig. 6D).

**Figure 7:**
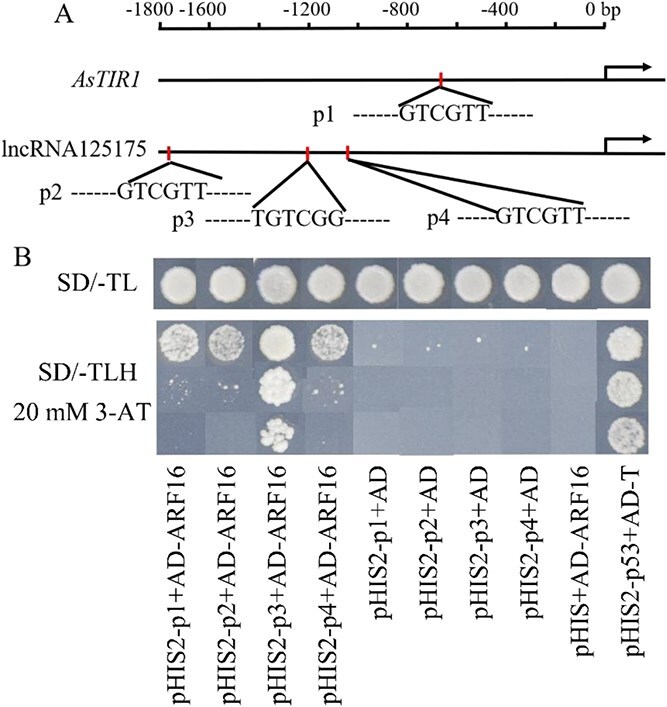
*AsARF* binds to the promoter of lncRNA125175. (A) Schematic diagram of pHIS2-P carrier construction. (B) Y1H assay of *AsARF* with the promoter of lncRNA125175/*AsTIR1*. AD, pGADT7 vector; pHIS2-p1/2/3/4 + AD and pHIS2 + AD-ARF16 was used as a negative control; pHIS2-p53, the vector contains the binding sequence of the p53 protein; AD-T, the vector expresses the SV40 Large T antigen and it can strongly bind to the p53 sequence. pHIS2-p53 + AD-T was used as positive control.

To further screen transcription factors that bind to the lncRNA125175/AsmiRNA393h/*AsTIR1* promoter, we performed gene co-expression network analysis using RNA-seq datasets (mRNA, lncRNA, and miRNA data) from different stages of garlic somatic embryogenesis (Fig. S3). More than 20 transcription factors, including ARF, WRKY, MYB, NAC, etc., were found to be co-expressed with lncRNA125175/*AsTIR1* in the same module. Based on the above analysis, it was found that the lncRNA125175 and *AsTIR1* promoters contained auxin-related *cis*-acting elements. Therefore, we focused on whether ARF (Asa8G04072) transcription factors could bind to the auxin *cis*-acting elements in the lncRNA125175 and *AsTIR1* promoters. To assess the importance of auxin-responsive elements for the transactivation of lncRNA125175 and *AsTIR1*, we divided the promoter into four fragments (p1–p4) for yeast one-hybrid (Y1H) assay (Fig. 7A). We found that AsARF16 specifically interacted with the p3 fragment, but not with p1, p2, or p4 (Fig. 7B). This finding indicated that the *cis*-element recognized by AsARF16 was located within the p3 region of the lncRNA125175 promoter, which contained a conserved auxin response element (AuxRE) with the core motif TGTCGG.

### AsARF16 regulated somatic embryogenesis development via interaction with AsWRKY31/AsIAA33

**Figure 8:**
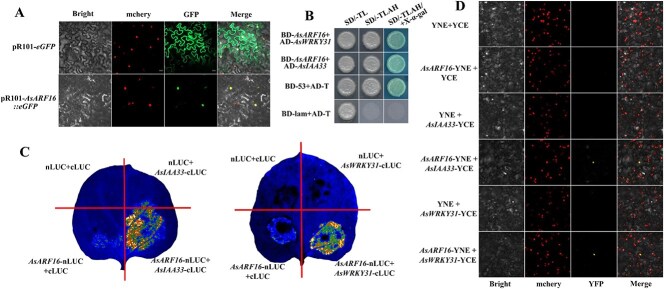
Subcellular localization and interaction verification of AsARF16. (A) Subcellular localization of *AsARF16*. (B) Y2H showed that the *AsARF16* interacted with AsWRKY31/AsIAA33 by growth on SD/-L-T-H-A + X-α-Gal plates. BD, pGBKT7 vector; AD, pGADT7 vector; BD-53, BD fused with p53 (positive control); BD-lam, BD fused with lamin C (negative control); AD-T, AD fused with the SV40 large T-antigen (positive control interaction partner for p53). (C) LCA exhibited that *AsARF16* interacted with AsWRKY31/AsIAA33, where the remaining combinations were used as negative controls. (D) BiFC analysis of AsARF16 and AsWRKY31/AsIAA33, where the remaining combinations were used as negative controls.

Expression level of *AsARF16* increased with 2,4-D application in the medium, suggesting that AsARF16 may play a crucial role during garlic somatic embryogenesis (Fig. S4). To further explore the molecular mechanism by which AsARF16 regulated somatic embryogenesis in garlic, proteins that may interact with AsARF16 were screened. Subcellular localization showed that AsARF16 was located in the nucleus (Fig. 8A). The AsWRKY31 (Asa2G07276) and AsIAA33 (Asa2G05881) were screened to potentially interact with AsARF16 through yeast two-hybrid test (Y2H) (Fig. 8B). The bimolecular fluorescence complementation (BiFC) and luciferase complementation assays (LCA) were further conducted in *N. benthamiana* leaves for verification. The fluorescence was detected in AsARF16-nYFP + AsIAA33-cYFP combination and AsARF16-nYFP + AsWRKY31-nYFP combination, as well as 771-nLUC-AsARF16 + 772-cLUC-AsIAA33 combination and 771-nLUC-AsARF16 + 772-cLUC-AsWRKY31 combination, which was not observed in negative controls (Fig. 8C, D). These results showed that AsARF16 interacted with AsWRKY31/AsIAA33 (Fig. 8C, D).

## Discussion

### LncRNA125175 served as an eTM for AsmiR393h to regulate *AsTIR1* during garlic somatic embryogenesis

Accumulating evidence demonstrated that long noncoding RNAs functioned as eTMs for miRNAs, effectively sequestering miRNAs and preventing their interaction with target mRNAs [27, 40, 41]. Chen *et al.* [42] predicted that lncRNA could be an eTM of miRNA in longan using TAPIR. Similarly, Zhang *et al.* [29, 30] predicted that lncRNA354 could be used as an eTM for miR160b in cotton using RNAhybrid. Building on these findings, our study focused on the lncRNA125175–AsmiR393h–*AsTIR1* module in garlic. We further predicted a circular protrusion structure between lncRNA125175 and the 9–11 bases at the 5′ end of AsmiR393h using TAPIR and RNAhybrid software (Fig. 2C).

Transient expression assays in maize and *N. benthamiana* have demonstrated that *PILNCR1* functions as an eTM by inhibiting ZmmiR399-guided cleavage of *ZmPHO2* [43]. Yang *et al.* [44] proved that MLNC4.6 was the eTM of miRNA156a by GUS staining. Here, functional validation through transient expression assays in *N. benthamiana* and garlic protoplasts demonstrated that lncRNA125175 acted as an eTM for AsmiR393h, thereby alleviating AsmiR393h-mediated repression of *AsTIR1* (Fig. 3). Transient overexpression of lncRNA125175 in garlic leaf protoplasts led to significantly increased *AsTIR1* expression and concurrently decreased AsmiR393h levels, showing that lncRNA125175 acted as an eTM of AsmiR393h to indirectly regulate *AsTIR1*.

In *Arabidopsis*, miR393 affected the sensitivity of explants to auxin by regulating the expression of auxin receptor TIR1 and AFB2, thus participating in inducing *Arabidopsis* somatic embryos [20]. The expression of lncRNA125175 and AsmiR393h showed the opposite trend in garlic somatic embryogenesis [39]. MiR393h was identified as the eTM of *TIR1* in cotton somatic embryogenesis, aligning with our results in garlic [21]. The F-box, Transp-inhibit, and AMN1 domains were found in different species with the same gene structure (Figs S5 and S6), indicating that *TIR* was highly conserved in different species. Here, AsmiR393h matures and *TIR1* had high consistency among different species (Fig. 2B), indicating that lncRNA125175 indirectly regulated *AsTIR1* and participated in somatic embryogenesis of garlic as an eTM of AsmiR393h.

### Auxin-mediated lncRNA125175–AsmiR393h–*AsTIR1* module regulated somatic embryogenesis

Auxin plays a pivotal role in somatic embryogenesis, and 2,4-D, a synthetic auxin analog, is extensively employed in the induction of somatic embryos [7]. Exogenous auxin modulates various aspects of plant growth and development by perturbing the dynamic equilibrium of endogenous auxin [45]. In plant species such as longan and spruce, the transition from nonembryogenic callus to embryogenic callus is accompanied by an elevation in endogenous IAA levels [45, 46]. Here, 2,4-D treatment effectively induced somatic embryogenesis, accompanied by marked alterations in auxin content (Fig. 5I). Moreover, auxin acts as a signaling molecule to regulate the expression of downstream target genes. The promoters of lncRNA125175 and *AsTIR1* contained three and one auxin-responsive elements, respectively, and their promoter activities were validated via GUS staining (Fig. 6D). Meanwhile, lncRNA125175 and AsmiR393h exhibited contrasting expression patterns under 2,4-D and NPA treatments, which was consistent with a competitive interaction centered on *AsTIR1* (Fig. 5I). Collectively, these findings demonstrated that the auxin-mediated lncRNA125175–AsmiR393h–*AsTIR1* regulatory module governed somatic embryogenesis in garlic.

Previously, co-expression cluster analysis had grouped lncRNA125175 and *AsTIR1* within the same module [39], which was found to be enriched for various transcription factors, including ARFs (Fig. S3). Given this co-expression pattern and the presence of AuxREs, we inferred that ARF could directly bind to these promoters. The Y1H assays suggested that an ARF protein specifically bonded to the second auxin response element within the lncRNA125175 promoter. The ARF members of the whole garlic genome were identified and named according to their position on the chromosome, among which AsARF16 was found to bind to the lncRNA125175 promoter (Fig. S7).

### The AsARF16–AsIAA33/AsWRKY31 transcriptional complex regulated somatic embryogenesis via the lncRNA125175–AsmiR393h–*AsTIR1* module

Our co-expression network analysis placed AsARF16, lncRNA125175, *AsTIR1*, and notably, AsIAA33 within the same regulatory module. This was highly suggestive, as ARF transcription factors and AUX/IAA repressors form the core module of the canonical auxin signaling pathway: AUX/IAA proteins bind to and inhibit ARFs in the absence of auxin; upon auxin perception, AUX/IAA proteins are degraded and then ARFs were released to activate downstream targets [47]. The AsARF16–AsIAA33 interaction shown in Fig. 8 validated the conservation of this core mechanism in garlic. More importantly, this ARF–IAA module was identified as the direct upstream regulator of the ‘lncRNA125175–AsmiR393h–*AsTIR1*’ module, thereby linking the classic transcriptional response to a novel layer of post-transcriptional regulation.

The complexity of this regulatory hub is further enriched by the inclusion of AsWRKY31, another member of the same co-expression module. WRKY transcription factors have been increasingly implicated in somatic embryogenesis across species, often in connection with auxin signaling [36–38]. For instance, *Arabidopsis* WRKY23 indirectly regulated somatic embryogenesis through the auxin pathway [34]. Our finding that AsWRKY31 physically interacted with AsARF16 suggested that AsWRKY31 likely functioned as a transcriptional co-regulator within this complex.

The formation of this AsARF16–AsIAA33/AsWRKY31 complex proposed a sophisticated regulatory model. The AsIAA33 component embedded the module within the conserved auxin-responsive feedback loop, ensuring rapid response to hormonal signals. Meanwhile, the incorporation of AsWRKY31 potentially enabled the integration of auxin signaling with other developmental or stress-related cues, a feature crucial for the precise spatiotemporal activation of somatic embryogenesis *in vitro*. This collaborative mechanism—where AsARF16 served as the core DNA-binding factor, AsIAA33 conferred signal sensitivity, and AsWRKY31 provided regulatory specificity or potency—allowed for the precise tuning of lncRNA125175 transcription. Consequently, the downstream ceRNA network was activated with the necessary strength and accuracy to drive the embryogenic transition.

## Materials and methods

### Plant materials

Garlic (*A. sativum* L.) cultivar ‘*Ershuizao*’ was obtained from the Vegetable Physiology and Ecology Laboratory at Nanjing Agricultural University, China. For somatic embryogenesis induction, we followed the protocol established by Bai *et al.* [39]. Explants at various developmental stages, including callus, embryogenic callus, and globular embryos, were collected based on morphological and histological characteristics. All samples were immediately frozen in liquid nitrogen and stored at −80°C for further analysis. Tobacco (*N. benthamiana*) plants were cultivated at controlled conditions (16 h/8 h, light/dark) for transient expression assays.

### Total RNA extraction and RT-qPCR

Total RNA isolation was performed using TRIzol™ Reagent (Invitrogen, USA). First-strand cDNA was synthesized from total RNA with HiScript IV 1st Strand cDNA Synthesis Kit (Vazyme, Nanjing, China). RT-qPCR analysis was carried out with TOROIVD qRT Master Mix (Toroivd, Shanghai, China). Gene expression levels were analyzed using gene-specific primers (Table S1). Gene expression levels were calculated using the 2^−ΔΔCt^ method with *AsBES* as control.

### 5′ RLM-RACE

5′ RLM-RACE was conducted as previously described by Yang *et al.* [21]. 5′ RNA adapter ligation with T4 RNA ligase followed by cDNA synthesis and PCR amplification using 5′ RNA adaptor primer and 3′ gene-specific primer was conducted.

### Histochemical GUS staining

For GUS staining assays, coding sequences of *AsTIR1*, AsmiR393h precursor, and lncRNA125175 were fused with pBI121-GUS vector. Tissues were vacuum infiltrated with staining solution (containing X-Gluc substrate) for 30 min, followed by incubation at 37°C for 2 h in the dark before observation. For transient expression in *N. benthamiana*, bacterial suspensions (OD_600_ = 0.4) in infiltration buffer (0.2% Triton-X-100, 50 mM NaPO_4_, 2 mM K_3_[Fe(CN)_6_], 2 mM K_4_[Fe(CN)_6_], and 2 mM X-Gluc) were pressure infiltrated into leaves using a needleless syringe.

### Transient expression in *N. benthamiana*

The recombinant plasmid was transformed into *Agrobacterium* strain GV3101. The bacterial suspension was prepared in an infiltration buffer containing 150 μM acetosyringone, 10 mM MgCl_2_, and 10 mM MES (pH 5.7), with the optical density adjusted to OD_600_ = 0.4 for agroinfiltration. After infecting the leaves, tobacco was incubated for 1 day under darkness followed by 2 days under light.

### Isolation and transformation of protoplasts from garlic leaves

Garlic protoplasts were isolated and transiently transformed following the established protocol of Peiter *et al.* [48]. Garlic leaf protoplasts were isolated using an enzymatic solution containing 0.5 M mannitol (Sigma, Germany), 0.5% macerozyme R10 (Yakult Pharmaceutical Ind. Co., Ltd., Japan), 2% cellulase R10 (Yakult Pharmaceutical Ind. Co., Ltd.), 20 mM KCl (Sigma), and 20 mM MES (pH 5.5). The solution was cooled to room temperature and added with 0.1% BSA (Sigma) and 10 mM CaCl_2_ (Sigma). The enzyme solution was sterilized via filtering through a 0.45-μm pore-size membrane filter. Tender garlic leaves were selected, and the leaves were immediately immersed in the enzymatic hydrolysate for 4 h in darkness. The enzymatic digestion was terminated by adding an equal volume of W5 solution (154 mM NaCl, 125 mM CaCl_2_, 5 mM KCl, 2 mM MES, pH 5.8). Protoplast yield was quantified using a hemocytometer, and viability was detected by FDA staining (2 μg/ml). The cells were cultured at 25°C overnight in darkness after transformation, which were then observed using a laser scanning confocal microscope.

### Y2H assays

Prey (*AD-AsWRKY31*/*AD-AsIAA33*) and bait (*BD-AsARF16*) vectors were constructed for Y2H analysis. The pGBKT7-53 + pGADT7-T plasmid combination served as the positive control. The AH109 yeast strain was utilized to conduct Y2H assays. The yeast transformation process was carried out in accordance with the detailed guidelines provided by the manufacturer. Transformants obtained on SD/-Trp/-Leu medium were cultivated on SD/-Trp/-Leu/-His/-Ade medium supplemented with X-a-galactosidase (X-a-gal) for growth.

### Y1H assays

The Y1H assays were performed with the Matchmaker Gold System (Clontech, Tokyo, Japan) following standard protocols. The full-length sequence of *AsARF16* was inserted into the pGADT7 vector by EcoRI and BamHI restriction sites to construct the prey plasmid. The promoter fragment of lncRNA125175 and *AsTIR1* were inserted into the pHIS2 vector by EcoRI and SmaI, which were named as pHIS2-P1, pHIS2-P2, pHIS2-P3, and pHIS2-P4. For negative controls, yeast strain Y1HGold was co-transformed with the empty pGADT7 vector and prey plasmid, followed by selection on SD/-TL medium containing gradient concentrations of aureobasidin A (AbA). The interaction between pGADT7-*AsARF* and pHIS2-P1/2/3/4 were tested using SD/-LTH/AbA medium.

### BiFC assays

pSPYNE and pSPYCE vectors were applied to examine the potential interactions between AsARF16 and AsWRKY31/AsIAA33. The coding sequences of AsARF16, AsWRKY31, and AsIAA33 were fused into vectors, respectively. Two plasmid combinations into *N. benthamiana* leaves including AsARF16-pSPYNE + AsWRKY31-pSPYCE and AsARF16-pSPYNE + AsIAA33-pSPYCE were introduced using *Agrobacterium*-mediated transient transformation. Empty vector combinations (pSPYNE + pSPYCE) served as negative controls.

### Firefly luciferase complementation imaging assays

To further validate the protein–protein interactions, we employed luciferase complementation imaging by fusing AsARF16 with 771-nLUC, while AsWRKY31 and AsIAA33 were individually fused with 772-cLUC. The recombination vectors (771-nLUC-AsARF16 + 772-cLUC-AsWRKY31, 771-nLUC-AsARF16 + 772-cLUC-AsIAA33, and 771-nLUC-AsARF16 + cLUC) were transiently expressed in tobacco leaves via *Agrobacterium* strain GV3101. After 2 days of incubation under darkness, leaves were treated with 15 mg/ml D-luciferin potassium salt (Sigma). Luciferase activity was quantified using a plant *in vivo* imaging system (PIXIS 1024B; Princeton Instruments, USA).

### Statistical analysis

The measurements included three biological replications. IBM SPSS Statistics 16.0 (SPSS Inc., Chicago, IL, USA) was applied to Student’s *t*-test and ANOVA (analysis of variation).

## Supplementary Material

Web_Material_uhag016

## Data Availability

The data underlying this article are available in the article and in its online supplementary material.
