# Supplementary material for: Auxin-induced AsARF16 complex orchestrates lncRNA125175-mediated ceRNA networks to regulate garlic somatic embryogenesis
Source: Hortic Res. 2026 Jan 20;13(4):uhag016. doi: 10.1093/hr/uhag016 (PMC13103477; doi:10.1093/hr/uhag016)
Supplement: Web_Material_uhag016 [file web_material_uhag016.zip › supplemental figures .docx]

**
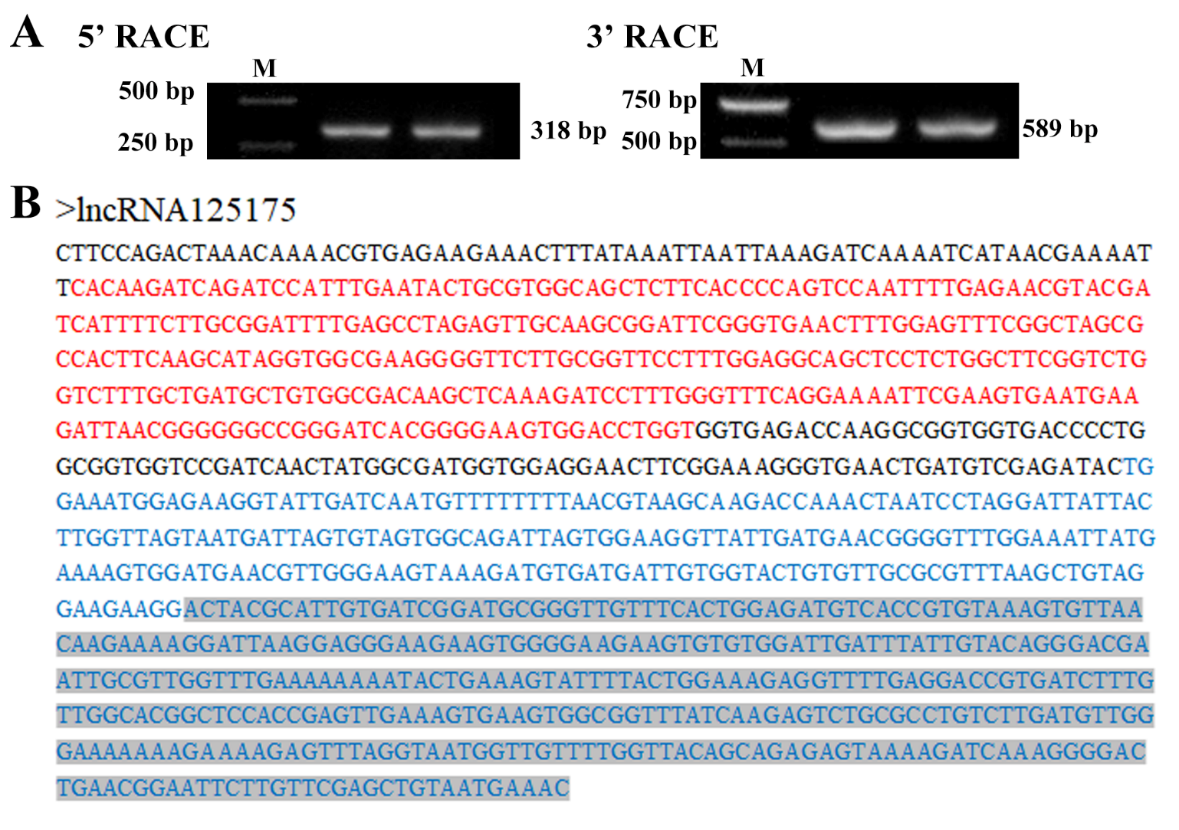
Figure. S1. Complete sequence identification of lncRNA125175.** (A) The left and right image showed 5’ and 3’ RACR amplification. (B) lncRNA125175 complete sequence splicing, where red and blue words indicated 5’ and 3’ RACE amplification

**
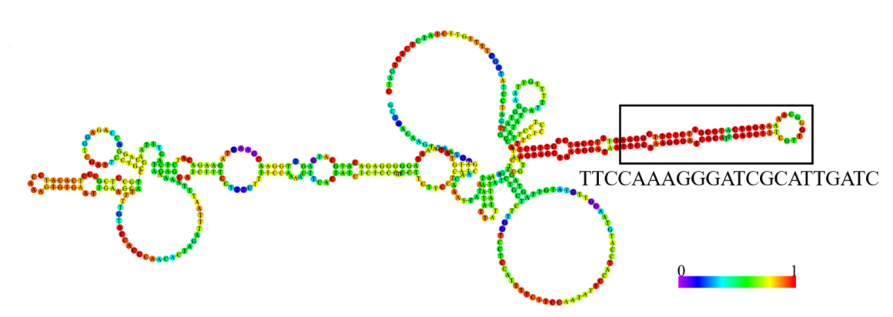
Fig. S2.** **Secondary structure of *AsmiR393h* precursor gene.**


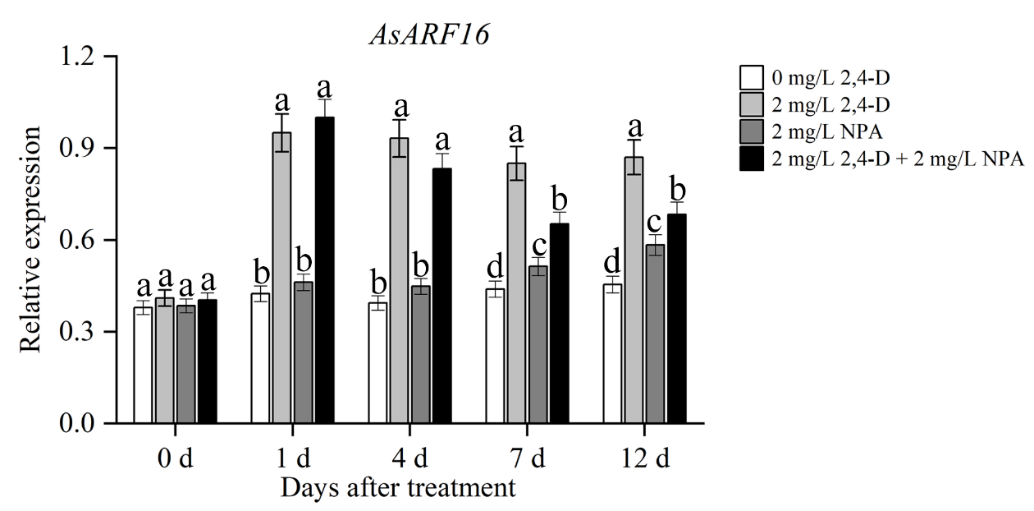
**Fig. S3.** **Changes of AsARF16 expression after 2,4-D and NPA.**


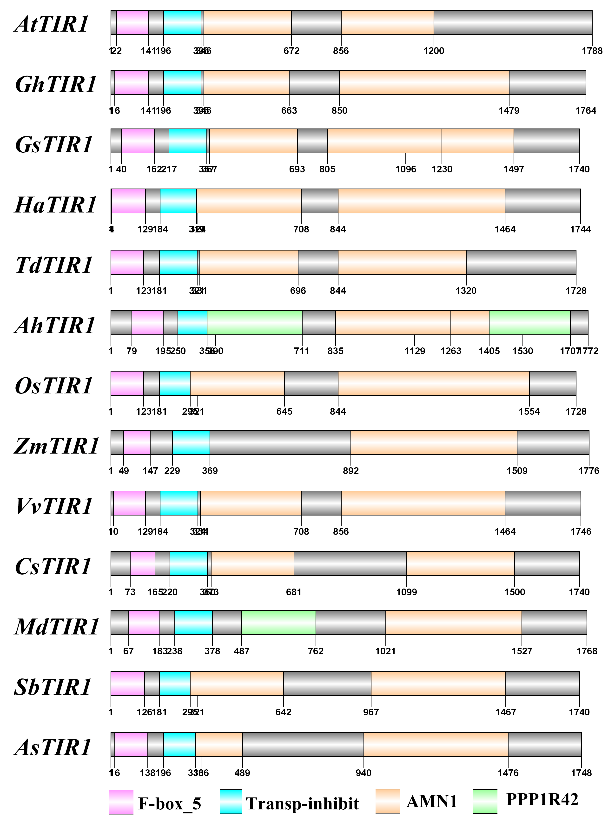
**Fig. S4.** **Analysis of the conserved structural domains of *AsTIR1.***

At: *Arabidopsis thaliana*; Gh: *Gossypium hirsutum*; Gs: *Glycine soja*; Ha: *Helianthus annuus*; Td: *Triticum dicoccoides*; Ah: *Arachis hypogaea*; Os: *Oryza sativa;* Zm: *Zea mays*; Vv: *Vitis vinifera*; Cs: *Citrus sinensis*; Md: *Malus domestica*; Sb: *Sorghum bicolor*; As: *Allium sativum*


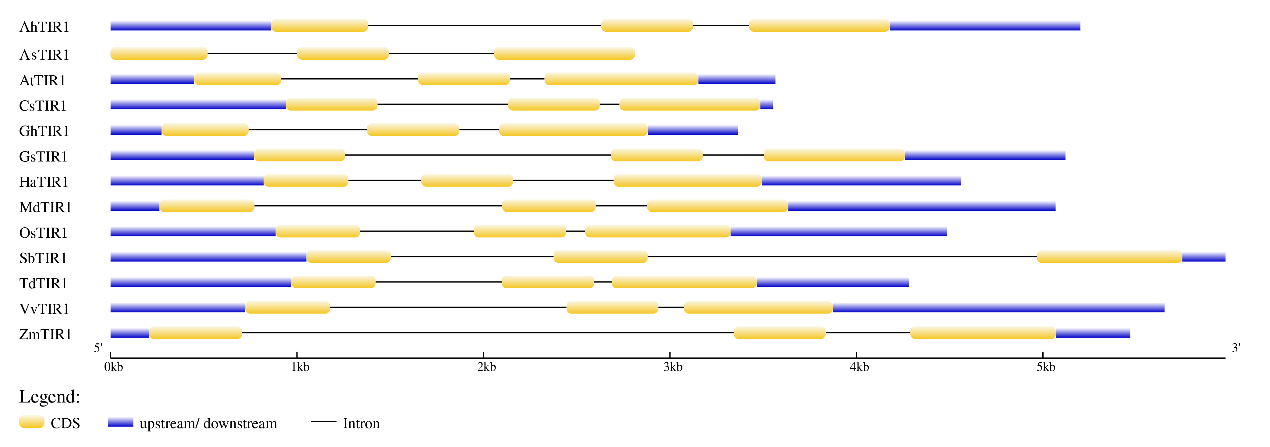
**Fig. S5. Analysis of *TIR1* genes structure in different species.** Ah: *Arachis hypogaea*; As: *Allium sativum*; At: *Arabidopsis thaliana*; Cs: *Citrus sinensis*; Gh: *Gossypium hirsutum*; Gs: *Glycine soja*; Ha: *Helianthus annuus*; Md: *Malus domestica*; Os: *Oryza sativa*; Sb: *Sorghum bicolor*; Td: *Triticum dicoccoides*; Vv: *Vitis vinifera*; Zm: *Zea mays*.


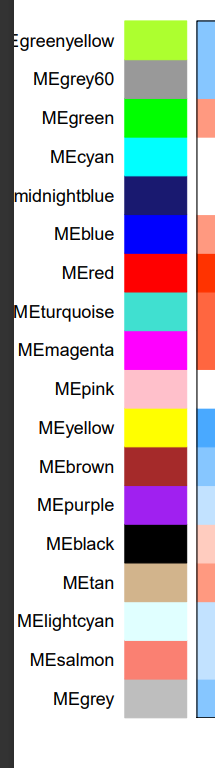

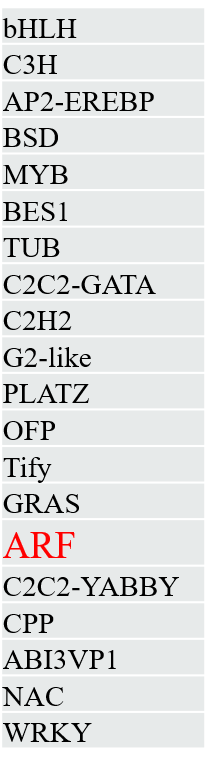


lncRNA125175

*AsTIR1*

**SS**

**Fig. S6. Transcription factor analysis of MEred gene module.**

**
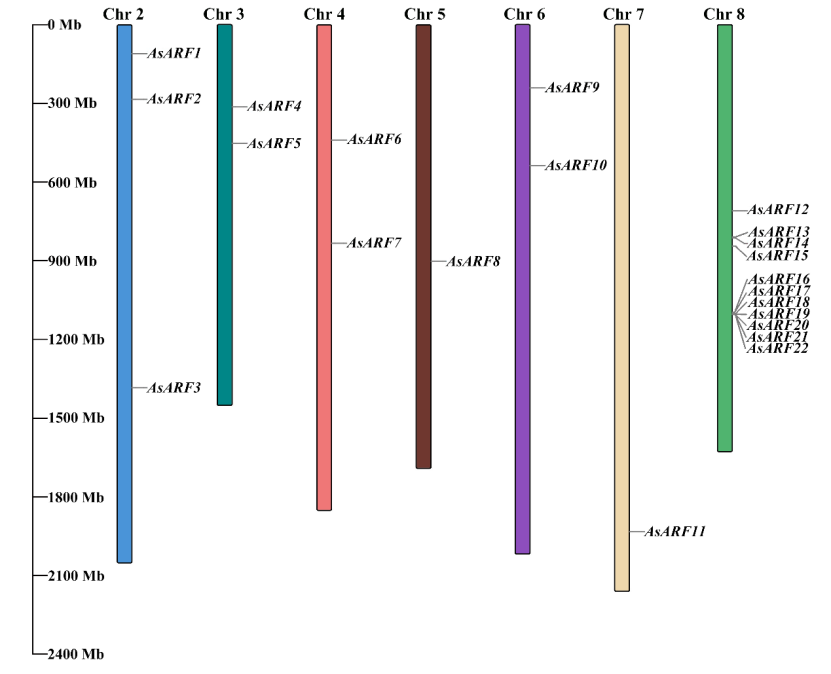
Fig. S7. The distribution of *AsARFs* genes on garlic chromoso**
