# Supplementary material for: Auxin-induced AsARF16 complex orchestrates lncRNA125175-mediated ceRNA networks to regulate garlic somatic embryogenesis
Source: Hortic Res. 2026 Jan 20;13(4):uhag016. doi: 10.1093/hr/uhag016 (PMC13103477; doi:10.1093/hr/uhag016)
Supplement: Web_Material_uhag016 [file web_material_uhag016.zip › supplemental tables.docx]

**Table S1. Primers used in the current study.**

| **Primer name** | **Sequence** | **Applicati-on** |
| --- | --- | --- |
| 35S::lncRNA125175-GUS-Fw | AGAACACGGGGGACTCTAGACACAAGATCAGATCCATTTG | GUS |
| 35S::lncRNA125175-GUS-Rv | ACGACGGCCAGTGAATTCGTTTCATTACAGCTCGAACAAG | GUS |
| 35S::ORF-GUS-Fw | AGAACACGGGGGACTCTAGAATGAAGATTAACGGGGGGCC | GUS |
| 35S::ORF-GUS-Rv | GACTGACCACCCGGGGATCCGTTCACCCTTTCCGAAGTTC | GUS |
| 35S::mORF-GUS-Fw | AGAACACGGGGGACTCTAGAGTGAAGATTAACGGGGGGCC | GUS |
| 35S::mORF-GUS-Rv | GACTGACCACCCGGGGATCCTCAGTTCACCCTTTCCGAAG | GUS |
| 35S::MIR393h-GUS-Fw | AGAACACGGGGGACTCTAGA | GUS |
| 35S::MIR393h-GUS-Rv | ACGACGGCCAGTGAATTC | GUS |
| 35S::*TIR*-GUS-Fw | AGAACACGGGGGACTCTAGAATGCAATTTACAACCATGAC | GUS |
| 35S::*TIR*-GUS-Rv | GACTGACCACCCGGGGATCCCAACGTCCATACAAAACCAG | GUS |
| 35S::mLncRNA-GUS-Fw | ATCTTCCGGTTGGTTTCCAGGCAGCTCCTCTGGCTTCGGT | GUS |
| 35S::mLncRNA-GUS-Rv | GGAAACCAACCGGAAGATCCCCTTCGCCACCTATGCTTGAAG | GUS |
| 0800-lncRNA125175-Fw | TCGACGGTATCGATAAGCTTGTGATTAAGCACTGAAATCACTTC | LUC |
| 0800-lncRNA125175-Rv | GCTCTAGAACTAGTGGATCCGCAGAAATCAAAAGGAGTTTGAG | LUC |
| 0800-*AsTIR1*-Fw | TCGACGGTATCGATAAGCTTCCCATATGGTCGACGTACTGA | LUC |
| 0800-*AsTIR1*-Rv | GCTCTAGAACTAGTGGATCCTCTCTATCACATCTCCATTCTGC | LUC |
| 0800-miR393h-Fw | TCGACGGTATCGATAAGCTTCTCAAAGAGTTCGCTGGAATG | LUC |
| 0800- miR393h -Rv | GCTCTAGAACTAGTGGATCCACTGTTGTGGTAATTATGGCTGAG | LUC |
| pHIS2-P1-Fw | ACTCACTATAGGGCGAATTCCGTGATTCCATCATTAATCT | Y1H |
| pHIS2-P1-Rv | GAACGCGTGAGCTCCCCGGGCTGCTCTTTATATGTTGAAC | Y1H |
| pHIS2-P2-Fw | ACTCACTATAGGGCGAATTCGTGATTAAGCACTGAAATCAC | Y1H |
| pHIS2-P2-Rv | GAACGCGTGAGCTCCCCGGGATTCCCTGGTTTTATTTCAA | Y1H |
| pHIS2-P3-Fw | ACTCACTATAGGGCGAATTCGAAGGTGAACTAATCAGGGA | Y1H |
| pHIS2-P3-Rv | GAACGCGTGAGCTCCCCGGGGCCAAGCGGCTAGCCCCATA | Y1H |
| pHIS2-P4-Fw | ACTCACTATAGGGCGAATTCGGACCTAGTTAGTAGGGTATG | Y1H |
| pHIS2-P4-Rv | GAACGCGTGAGCTCCCCGGGCGTTCATATTGCGCTGAGAT | Y1H |
| AD-*AsAR16F*-Fw | CCATGGAGGCCAGTGAATTCATGGCTCTTTCTGCTGCTAATC | Y1H |
| AD-*AsARF16*-Rv | CAGCTCGAGCTCGATGGATCCGCAATCCTTATCAGATGCAA | Y1H |
| BD-*AsARF16*-Fw | ATGGCCATGGAGGCCGAATTCATGGCTCTTTCTGCTGCTAATC | Y2H |
| BD-*AsARF16*-Rv | CGCTGCAGGTCGACGGATCCGCAATCCTTATCAGATGCAA | Y2H |
| AD-*AsARF16*-Fw | CCATGGAGGCCAGTGAATTCATGGCTCTTTCTGCTGCTAATC | Y2H |
| AD-*AsARF16*-Rv | CAGCTCGAGCTCGATGGATCCGCAATCCTTATCAGATGCAA | Y2H |
| AD-*AsIAA33*-Fw | CCATGGAGGCCAGTGAATTCATGGTGGGAGGTATTGAATTTGAG | Y2H |
| AD-*AsIAA33*-Rv | CAGCTCGAGCTCGATGGATCCCATAATTCGTAACCTCTTGCAAG | Y2H |
| AD-*AsWRKY31*-Fw | CCATGGAGGCCAGTGAATTCATGGAAAAGACCAGTGCAG | Y2H |
| AD-*AsWRKY31*-Rv | CAGCTCGAGCTCGATGGATCCTGCTGCTGTTGTTGAGAGG | Y2H |
| PR101-*AsARF16*-Fw | CTTCACTGTTGATACATATGATGGCTCTTTCTGCTGCTAATC | Subcellular localization |
| PR101-*AsARF16*-Rv | CCTTGCTCACCATGGATCCGCAATCCTTATCAGATGCAA | Subcellular localization |
| lncRNA125175  -PR101-Fw | GTTCTTCACTGTTGATACACAAGATCAGATCCATTTG | Overexpression |
| lncRNA125175  -PR101-Rv | CTCGCCCTTGCTCACCATGTTTCATTACAGCTCGAACAAG | Overexpression |
| *AsMIR393h*  -PR101-Fw | GTTCTTCACTGTTGATAGATCAGAGAGATAGAACAAAAG | Overexpression |
| *AsMIR393h*  -PR101-Rv | CTCGCCCTTGCTCACCATGCTAACAAGTGAATGTGATG | Overexpression |
| *AsTIR1*-PR101-Fw | GTTCTTCACTGTTGATAATGCAATTTACAACCATGACTTAC | Overexpression |
| *AsTIR1*-PR101-Rv | CTCGCCCTTGCTCACCATCAACGTCCATACAAAACCAGG | Overexpression |
| 5’ GSP | ACCAGGTCCACTTCCCCGTGATCCCG | 5’ GSP |
| 3’ GSP | TGGAAATGGAGAAGGTATTGATCAATG | 3’ GSP |
| N-Bifc-*AsARF16*-Fw | AACACGGGGGACTCTAGAATGGCTCTTTCTGCTGCTAATC | BIFC |
| N-Bifc-*AsARF16*-Rv | CTTTTGCTCCATCCCGGGTCAGCAATCCTTATCAGATGCAA | BIFC |
| N-LUC-*AsARF16*-Fw | CTCGGTACCCGGGGATCCATGGCTCTTTCTGCTGCTAATC | LCA |
| N-LUC-*AsARF16*-Rv | GTACGAGATCTGGTCGACTCAGCAATCCTTATCAGATGCAA | LCA |
| C-BIFC-*AsWRKY31*  -Fw | AACACGGGGGACTCTAGAATGGAAAAGACCAGTGCAG | BIFC |
| C-BIFC-*AsWRKY31*  -Rv | GTATGGGTACATCCCGGGTGCTGCTGTTGTTGAGAGG | BIFC |
| C-LUC-*AsWRKY31*-Fw | GCGTCCCGGGGCGGTACCATGGAAAAGACCAGTGCAG | LCA |
| C-LUC-*AsWRKY31*-Rv | AGTCCATTTGTTGGATCCTGCTGCTGTTGTTGAGAGG | LCA |
| C-BIFC-*AsIAA33*-Fw | AACACGGGGGACTCTAGAATGGTGGGAGGTATTGAATTTGAG | BIFC |
| C-BIFC-*AsIAA33*-Rv | GTATGGGTACATCCCGGGCATAATTCGTAACCTCTTGCAAG | BIFC |
| C-LUC-*AsIAA33*-Fw | GCGTCCCGGGGCGGTACCATGGTGGGAGGTATTGAATTTGAG | LCA |
| C-LUC-*AsIAA33*-Rv | AGTCCATTTGTTGGATCCCATAATTCGTAACCTCTTGCAAG | LCA |
| miR393h-Fw | AAAATGGAGGAGCAAGAGCA | qRT-PCR |
| mir393h-Rv | ACATGCATGAGTTGGAACGA | qRT-PCR |
| *AsTIR1*--Fw | GGGAAACCTCATTTTGCTGA | qRT-PCR |
| *AsTIR1*-Rv | TCGGAAAAGAACGAGCAAGT | qRT-PCR |
| lncRNA125175-Fw | CGGGTTGTTTCACTGGAGAT | qRT-PCR |
| lncRNA125175-Rv | CCAACAAAGATCACGGTCCT | qRT-PCR |
| *AsARF16*-Fw | ATATTCCGTGGTCAGCCAAG | qRT-PCR |
| *AsARF16*-Rv | TGTTGCAAGGACACCAATGT | qRT-PCR |
| *AsBES*-Fw | GTATGGGCTTGGTTGGTAGG | qRT-PCR |
| *AsBES*-Rv | GTCGTTGAGCAGGATGGT | qRT-PCR |
